# Establishing Global School Feeding Program Targets: How Many Poor Children Globally Should Be Prioritized, and What Would Be the Cost of Implementation?

**DOI:** 10.3389/fpubh.2020.530176

**Published:** 2020-12-02

**Authors:** Lesley J. Drake, Nail Lazrak, Meena Fernandes, Kim Chu, Samrat Singh, David Ryckembusch, Sara Nourozi, Donald A. P. Bundy, Carmen Burbano

**Affiliations:** ^1^Partnership for Child Development, Imperial College, London, United Kingdom; ^2^World Food Programme, Rome, Italy; ^3^London School of Hygiene and Tropical Medicine, London, United Kingdom

**Keywords:** schoolchildren, adolescents, school health, school feeding, poverty, child development, human capital, nutrition

## Abstract

The creation of Human Capital is dependent upon good health and education throughout the first 8,000 days of life, but there is currently under-investment in health and nutrition after the first 1,000 days. Working with governments and partners, the UN World Food Program is leading a global scale up of investment in school health, and has undertaken a strategic analysis to explore the scale and cost of meeting the needs of the most disadvantaged school age children and adolescents in low and middle-income countries globally. Of the 663 million school children enrolled in school, 328 million live where the current coverage of school meals is inadequate (<80%), of these, 251 million live in countries where there are significant nutrition deficits (>20% anemia and stunting), and of these an estimated 73 million children in 60 countries are also living in extreme poverty (<USD 1.97 per day). 62.7 million of these children are in Africa, and more than 66% live in low income countries, with a substantial minority in pockets of poverty in middle-income countries. The estimated overall financial requirement for school feeding is USD 4.7 billion, increasing to USD 5.8 billion annually if other essential school health interventions are included in the package. The DCP3 (Vol 8) school feeding edition and the global coverage numbers were launched in Tunis, 2018 by the WFP Executive Director, David Beasley. These estimates continue to inform the development of WFP's global strategy for school feeding.

## Introduction

The World Food Program (WFP) is the United Nations lead organization on school feeding. Recent analyses indicate that the world has underinvested in the health and nutrition of school age children and adolescents, especially in low and middle-income countries, with negative consequences for the creation of human capital. WFP is therefore aiming to increase global investment in school feeding and school health, and has undertaken, with partners, a high-level analysis of the scale of need, to enhance the precision of strategic planning.

### A Paradigm Shift in Thinking About Human Development

It is now recognized that a major constraint on global development is the current under-investment in school age children and adolescents. A series of analyses published since 2017 have emphasized that there is a need to invest in child health, nutrition, and education throughout the first 8,000 days of life if children are to grow up to fulfill their potential as adults (1–3). Schools are the key to delivering these school health, school feeding, and education interventions, and thus serve as critical enabling platforms for the creation of human capital. The UN World Food Program is reimagining its role in this new vision of development (4), and in this publication sets out the way it is estimating the scale and scope of the interventions that it should provide to support the children most in need.

Investing in human capital—the sum of a population's health, skills, knowledge, and experience—can strengthen a country's competitiveness in a rapidly changing world (3). Child health and learning are critical to human capital development. A well-nourished, healthy, and educated population is the foundational pre-requisite for growth and economic development. A key contributor to the ranking in the Human Capital Index published by the World Bank is the quality of learning in a country, as measured by the new metric *Learning Adjusted Years of Schooling* (LAYS), which measures not only the amount of schooling, but also the quality of learning (5). School feeding can have a positive impact on LAYS through increasing attendance, particularly of girls, and by improving learning. Low-income countries in Africa have potentially the most to gain from school feeding since they represent 25 out of the 30 countries with the lowest Human Capital Index rankings. For many of these countries, underinvestment in human capital leads to a loss of economic potential, ranging from 50 to 70 percent in the long-term. Africa's Human Capital Index score puts the region at 40% of its potential, which implies that Africa's GDP could be 2.5 times higher if the benchmarks for health and education were achieved.

The 2017 3rd edition of the World Bank *Disease Control Priorities (DCP3)*, supported by the Gates Foundation, provides a new perspective on investing in child development (1). In particular, Volume 8, entitled *Child and Adolescent Health and Development*, confirms the importance of investing in the first 1,000 days of life, and also highlights the need to continue investment during key periods for development during the next 7,000 days, or until the early twenties. These findings have led to a move toward a new 8,000 days paradigm. Just as babies are not merely small people, they need special and different types of care from the rest of us, so growing children and adolescents are not merely short adults. They too have critical phases of development that need specific interventions, especially in the phases of pre-puberty, puberty, and during late adolescence.

The important role of schools in investing in children was emphasized by the UN Standing Committee on Nutrition in 2017, in a statement entitled *Schools as a System to Improve Nutrition*, which emphasizes the importance of school health and school feeding (2). Similarly, a publication prepared by the World Bank and the Global Partnership for Education entitled *Optimizing Education Outcomes: High-Return Investments in School Health for Increased Participation and Learning* (6), took this a step further, emphasizing the need to fix the almost complete mismatch between investments in the health of children, currently almost all focused on children under 5 years of age, and investment in education, mostly between 5 and 20 years of age.

### Crucial Investments in Children and in Human Capital

*Disease Control Priorities* Volume 8 (1) lists several elements of an essential package, including simple and cheap health interventions that promote education outcomes, such as deworming, correcting refractive errors (e.g., myopia, astigmatism, and hypermetropia), and malaria prevention. Within this essential package, school feeding is the most costly component, on an annual basis, essentially due to the fact that meals are delivered to children more frequently than any other intervention of the package, but is never-the-less *cost-effective* due to the multiple benefits it delivers. A recent Benefit-Cost Analysis (7) shows that school feeding programs could have substantial benefits for the costs invested, with about $20 of returns for $1 invested in school feeding programs, a return on investment comparable to several of the best-buy interventions analyzed by the Copenhagen Consensus exercise^1^. The large scale of benefits reflects the additive returns on investment from multiple sectors (8). For example, the analysis examined the returns in 14 low- and middle-income countries, and showed average Benefit-Cost Ratios of 13.5 to education (through human capital), 6.7 to the local economy (through local procurement and local employment), and 0.8 to social protection (the externality effect of the social safety net) and to health (7). Other potentially substantial and additional returns, for example to gender and peace-building, have yet to be quantified (4).

The World Bank's State of Social Safety Nets 2018 and the underlying ASPIRE database show that whilst school feeding is not the largest safety net worldwide (in terms of beneficiary numbers), it is the most widespread (in terms of number of countries). This highlights that not only has school feeding emerged as the main intervention for children in school, but also as the most widespread safety net worldwide regardless of the beneficiaries' category or age group.

Number of beneficiaries by category of safety net^*^ (sorted by decreasing order):

- Fee waivers: 382 M people
- School feeding: 357 M people
- Food and in-kind aid: 282 M people
- Unconditional cash transfers: 278 M people
- Conditional cash transfers: 185 M people
- Public works: 103 M people
- Social pensions: 83 M people

^*^The categories of social safety nets listed here are based on the World Bank's *State of Social Safety Nets 2018*. Fee waivers encompass health insurance exemptions and reduced medical fees, education fee waivers, food subsidies, housing subsidies and allowances, utility and electricity subsidies and allowances, agricultural inputs subsidies, and transportation benefits.

Number of countries which have a safety net, by category (sorted by decreasing order):

- School feeding: 116 countries
- Unconditional cash transfers: 90 countries
- Public works: 81 countries
- Food and in-kind aid: 77 countries
- Fee waivers: 65 countries
- Social pensions: 64 countries
- Conditional cash transfers: 60 countries

In real-world practice, school feeding has emerged as the main intervention for children in schools around which other elements, such as deworming or supplementation are delivered. Almost every country in the world provides food to its school children in some scale, in 2013 reaching about 368 million children worldwide (9).

When linked to good nutrition and education, well-designed equitable school feeding programs contribute to child development through increased years of schooling, better learning, and improved nutritional status (10, 11). School feeding provides consistent positive effects on energy intake, micronutrient status, school enrolment, and attendance of children (12–14). The effects are particularly strong for girls. In its influential 2016 report, *The International Commission on Financing Global Education Opportunity*, chaired by Gordon Brown, identified 13 nonteaching interventions as “highly effective practices to increase access and learning outcomes,” these included three health-related programs: school feeding, malaria prevention, and micronutrient intervention. A recent UN agency review of evidence finds that school feeding is one of the two interventions with the strongest evidence of impact on equity and inclusion (the other one being conditional cash transfers) (11).

School feeding is one of the most common safety nets (15, 16), providing the daily support and stability that vulnerable families and children need, and was shown to be one of the first social protection solutions that poor countries turned to during the social shocks of the 2008 financial crisis (13, 17). Finally, well-designed school feeding programs that procure food locally may offer major additional benefits, including an increased dietary diversity, new employment opportunities for women and/or smallholder farmers, and improved livelihoods for the local communities. Well-designed school feeding programs can specifically contribute to women's economic empowerment and decision-making by deliberately engaging women farmers and traders, and providing job opportunities to women in school canteens (4, 14).

This “new-generation” vision of school feeding has led the World Food Program to ask not only whether more can be done to support school feeding in low and middle-income countries, but also to try and determine which groups should be prioritized as most in need, and what would be the scale of need and the cost implications. These are real-world questions about the world today, which will shape the new global school feeding strategy of WFP. This paper shares the approach that WFP has used in answering these questions, in order to encourage understanding and to stimulate debate.

## Methods and Results

This section explains the methodology used by addressing a series of questions which indicate the sequential methodological steps used to estimate the scale of need and the cost of addressing the need. The first step was to estimate the number of children enrolled in school, and the number of children in schools currently covered by school feeding programs. The next step was to estimate the scale and location of the population of school children that are not covered by a school feeding program. This was followed by applying a sequence of indicators as filters to identify those most in need of school health and school feeding programs. Finally, published benchmarks were used to estimate the cost of reaching the population as identified most in need based on the previous step.

### How Many School Children Are There in Low- and Middle-Income Countries Globally, and How Many Receive School Meals?

UNESCO, the United Nations lead on Education is a ready source for regularly updated estimates of the numbers of school children in low and middle-income countries (18). Remarkably, there is no single global source that records how many of them receive school meals, or benefit from school health programs. The World Bank SABER (Systems Approach for Better Education Results) tool (19) potentially could provide an answer, and the World Bank is currently updating and revising its SABER tools to better fulfill this function.

In 2013, WFP led the first coherent attempt to address this question (9) and is establishing a continuous monitoring process, but for now, we have to rely on multiple different sources, from various dates, to build up a picture. There are four main sources:

- the WFP publication State of School Feeding Worldwide, which has been published once, in 2013, and which reports data from national statistics and the WFP country offices (9);
- the WFP publication *Smart School Meals: Nutrition-Sensitive National Programs in Latin America and the Caribbean*; published in 2017, this reports data on national programs in the LAC region (20);
- the World Bank publication: *The State of Social Safety Nets*, 2018 edition (16);
- the African Union publication: *Sustainable School Feeding*, published in 2018 (21).

**Figure 1:**
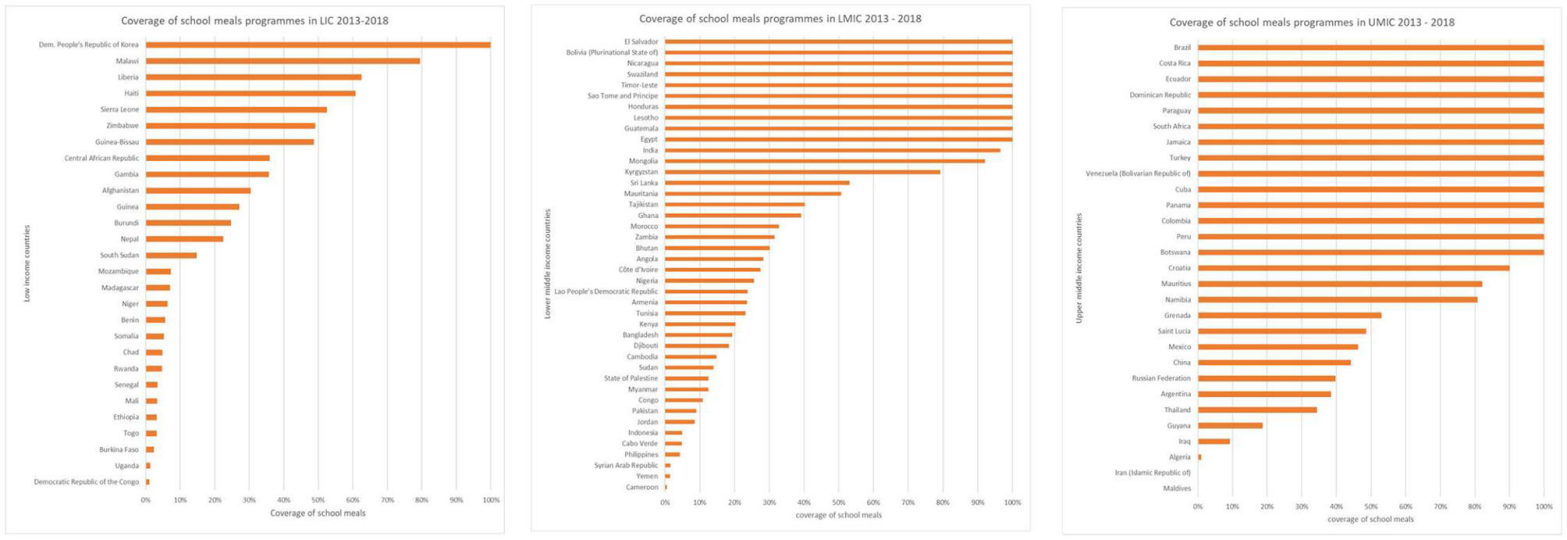
Proportion of children who receive school meals by country and income level.

The data suggest that there are 663 million school children enrolled in low- and middle-income countries globally. The countries vary in the number and proportion of these children who receive school meals (see Figure 1).

At least 305 million children are estimated to be fed at school every day of the school year. This suggests that 54% of the 663 million children enrolled in school in low- and middle-income countries do not currently receive meals at school. The key question going forward is what proportion of these children would benefit most from making these meals available.

### A Scoping Exercise: What Is the Likely Scale of Need, and What Indicators Are Available to Identify Populations in Need?

In order to refine the filters for identifying targets, we first explored which indicators might best be used to define population in need. The purpose of this preliminary step was to provide an order of magnitude estimate of the need for school feeding globally, before proceeding to more refined analysis. This order of magnitude is comprised of several estimates, calculated by applying various indicators to the same numerator: the number of children enrolled in schools in low- and middle-income countries (663 million children). Some of these indicators aimed to capture the scale of need for school feeding (metrics of extreme poverty, acute food insecurity, and chronic hunger), while others reflected the capacity of national governments and the international community to respond to that need (ranking against the published DCP3 benchmarks, which are described further below, and formal declaration of a national emergency).

These estimates were first calculated at the country level: for example, by using the published poverty rates, which vary between countries. The poverty rate of each country (expressed as the percentage of the population living in poverty for that country) is first applied to the school-going population of that country (expressed as the number of children going to school) to estimate the number of poor schoolchildren in that country. Country-level estimates are then summed across income groups to generate an estimate of poor schoolchildren in low- and middle-income countries.

The “DCP3 benchmarks” correspond to a hypothetical scenario proposed by the World Bank DCP3 publication as a realistic target for governments in the medium term (1). This target would be to provide coverage to 20% of school-going children in low-income countries and 40% of schoolchildren in middle-income countries. The DCP3 publication suggests that these percentages represent the minimum proportion of school-going children that would need health and nutrition support to successfully complete their education (1). If the current levels of coverage were found to be lower than these percentages then they were deemed insufficient to meet the needs of that population.

The analysis explored six indicators:

1. The benchmark in Disease Control Priorities (1): 20% of all school children in LICs and 40% in MICs.
2. The World Bank extreme poverty threshold ($1.90/day) (22).
3. The FAO estimates of chronic hunger (percentage of undernutrition) (23).
4. A combination of both the poverty and hunger metrics above.
5. The International Phase Classification and estimates of people living in acute food insecurity (IPC 3 and above)” (24).
6. Countries with a declared L2 or L3 emergency^*^ (25).

The results of these analyses for the first 4 indices are shown in Table 1, which indicates that the scale of all these estimates is remarkably similar at around 40–50 million children.

**Table 1:** Number of children in need of school feeding, and number of countries; based on different indicators of need [sources (1, 22, 23)].

**Indicator**	**Children in need (millions)**
DCP3 benchmarks: 20% LICs, 40% MICs	57.2
World Bank poverty threshold	43.7
FAO undernutrition metrics	22.6
Combined poverty and undernutrition metrics	51.0

^*^L2 and L3 refer to the two superior levels of emergency classification in a three-level scale defined by WFP as per *WFP Emergency Response Classifications, 2014*.

**Figure 2:**
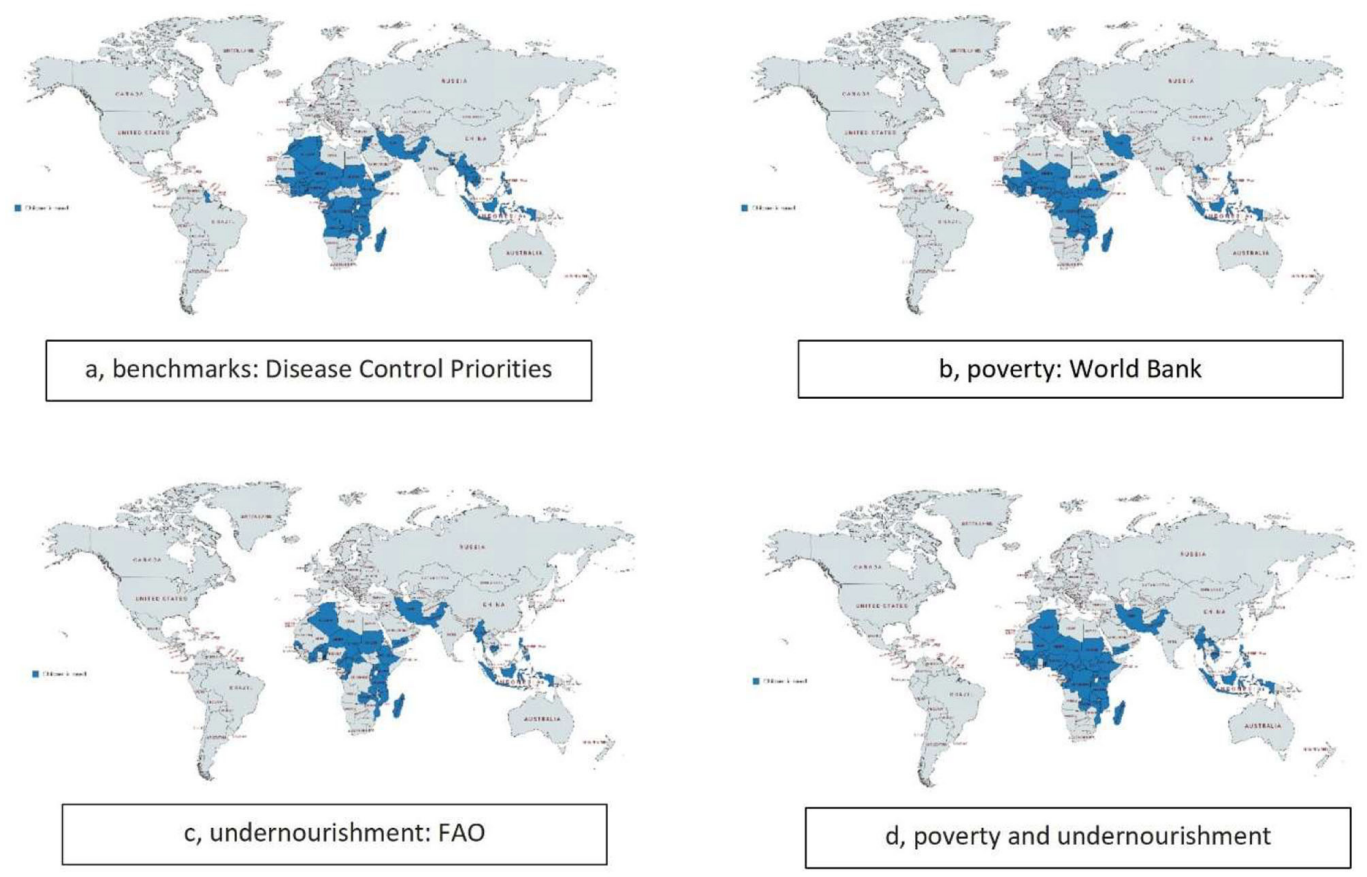
Distribution of children in need of school feeding in low- and middle-income countries globally.

Mapping the countries in which these children are found, Figure 2, also suggests broad similarities, with most of the at risk populations clustered in Africa, with some in South and South East Asia, but few elsewhere. The poverty and hunger indicators unsurprisingly suggest considerable overlap of these two conditions, and also demonstrates that combining the two indicators helps broaden the safety net, and includes more children at risk.

The final two indices which were considered, IPC and L2/3, are intended as real-time measures of emergency need. Unsurprisingly, these turn out to be highly geographically focused measures that, in the years examined, suggested the in-need populations of school age children were of the order of 1–6 million (data not shown). While these are large populations in terms of mobilizing emergency care, they are clearly underestimates of the scale of the populations of children with long-term developmental needs. IPC and L2/3 are appropriate indicators for identifying operational targets at the country level, but too narrow for the present purpose, and were accordingly not pursued.

### Which Current National School Feeding Programs Have Sub-optimal Coverage?

The analyses above indicate that there are 663 million children enrolled in schools in low- and middle-income countries, and that 305 million of these children receive school meals. Note that this figure excludes the ~13 million children who currently receive meals from WFP operations in these countries, as a key purpose of this exercise is to identify the role of other partners. The data does not show which children are targeted, and it is at least probable that many programs are regressive and a majority of these children are from the most affluent segments of the population. To develop a benchmark for addressing this question, the analysis considered the targets used by actual programs. For example, the long-standing and successful national program in South Africa, CSTL (*Care and support for teaching and learning)* targets the lower three quintiles of the school population, that is, 60% of the total population (26).

Taking a conservative view, it is assumed here that a school-feeding program which covers the lower four quintiles (that is, 80%) will likely ensure that all children in need are fed. Hence a target was set at 80% coverage (total number of children in school/number fed), a level which is taken to indicate confidence that coverage is already reaching most children in need, while populations below this threshold should be explored further. The reported coverage by country is shown in Figure 1. Using that cut-off, the population in need is reduced to the 328 million school children living in countries with sub-optimal access (<80% coverage) to school meals programs.

### Where Is School Feeding Most Likely to Make a Difference?

**Figure 3:**
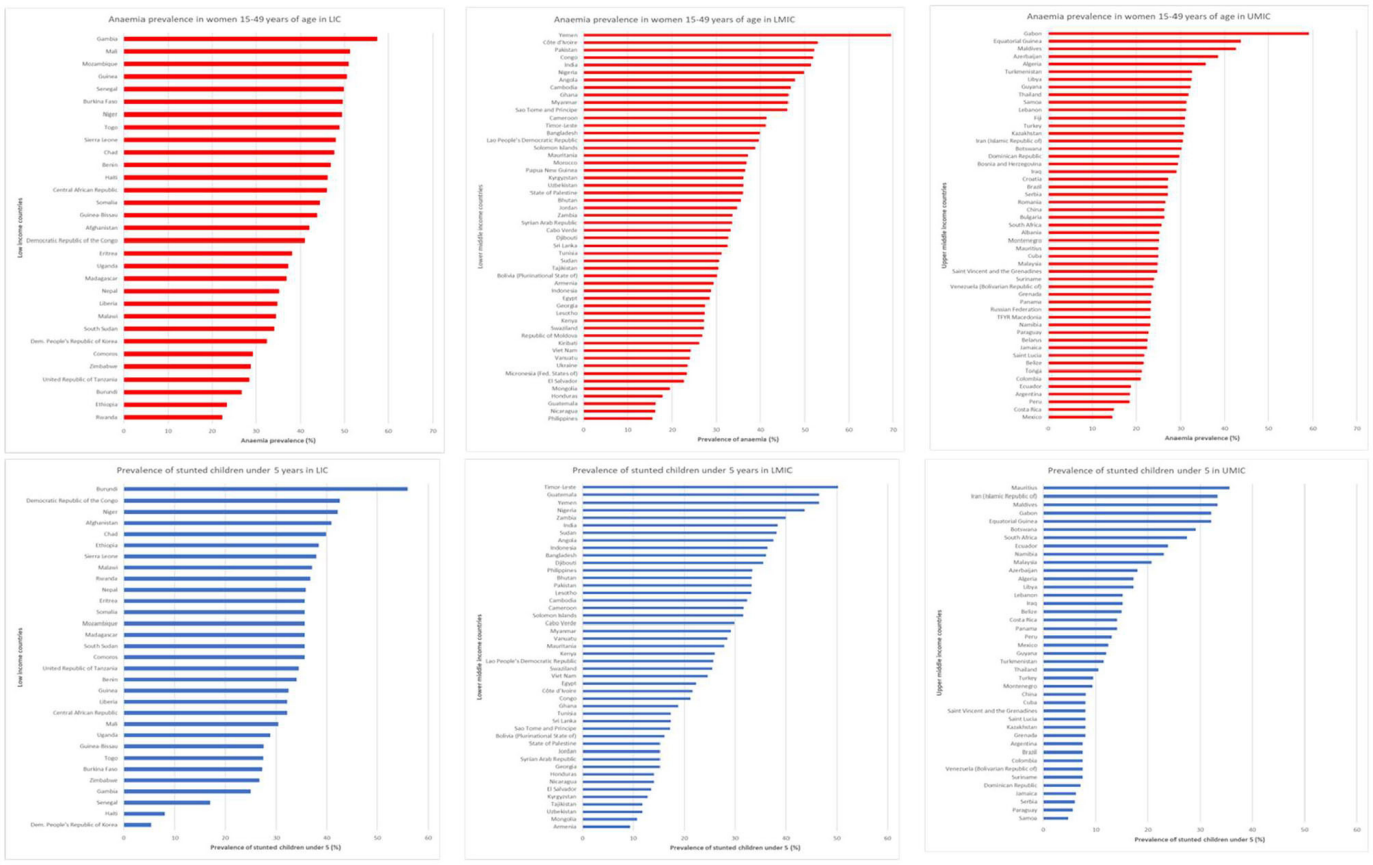
Prevalence of anemia (in women of reproductive age) and stunting (in children <5 years), by country, and income level [data from (23)].

Providing food and nutrition sensitive interventions is likely to be most effective where undernutrition is prevalent. The scoping exercise has also shown, using the FAO undernutrition measure (23, 27), that nutrition indicators could add a useful dimension to the targeting. In order to enhance the precision of this approach, and to use nutrition metrics which are generally available in countries, the two commonly available metrics of prevalence of anemia and stunting were adopted. No health indicator is collected regularly from the target school-age children or from adolescents, so the analysis used the standard reported metrics of prevalence of anemia in women of reproductive age, which is routinely collected at antenatal clinics, and prevalence of stunting in children <5, which is routinely collected as part of child health surveillance (27). The former is an indicator of current dietary lack, and the latter integrates undernutrition over time. These data are shown in Figure 3.

Using these filters, the target population was further refined to the 251 million who live in communities where anemia and stunting were existing challenges.

### Community Resilience and Journey to Self Reliance

Development investments are most effective in the long-term if they help countries to develop sustainable programs, as has been widely acknowledged by the humanitarian and development community, following the 2016 World Humanitarian Summit, and WFP is committed to this objective. But the target children of most immediate and greatest need are those least able to progress to self-reliance, or those for whom that journey has just begun.

**Figure 4:**
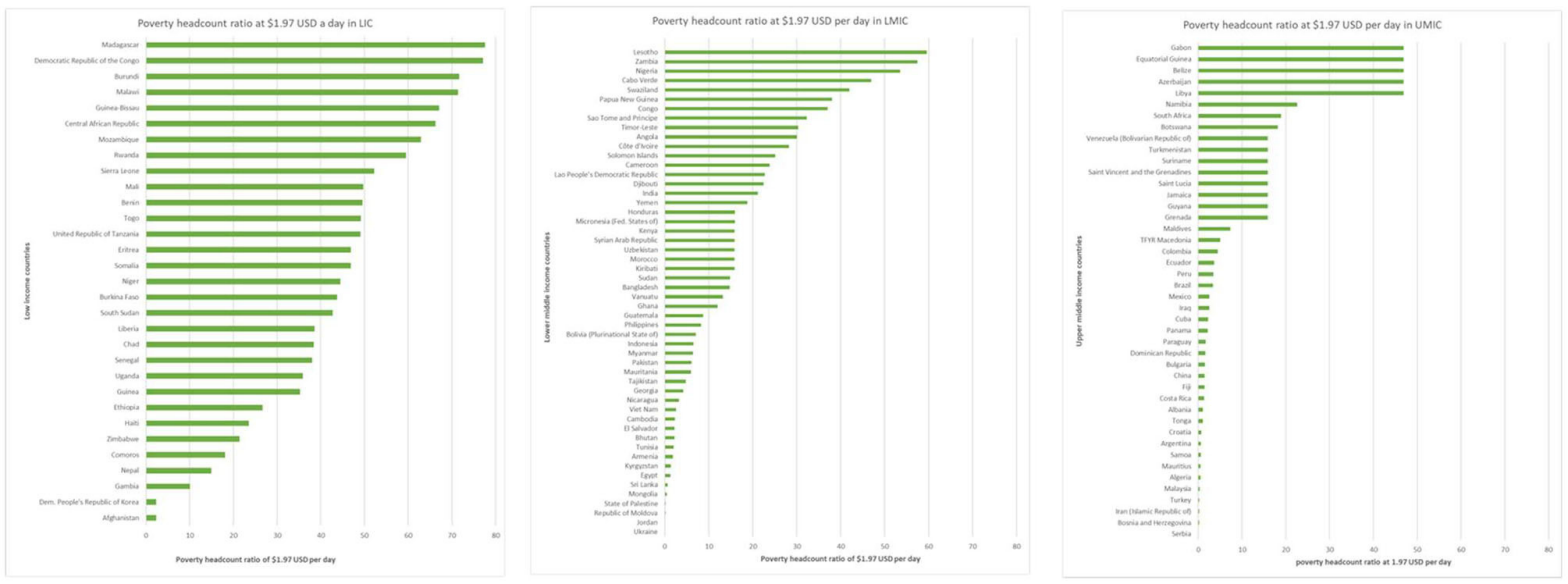
Proportion of populations living in extreme poverty, by country and income level (22).

WFP advocates for the universal adoption of school feeding programs and is committed to supporting all governments develop their national school feeding programs. However, a line must be drawn between communities which, despite their limited resources, have reached a certain level of resilience to support their own needs for school feeding, and those in which immediate needs exceed their current capacity and require public institutions, such as their governments and/or international agencies, to provide them with social assistance and school feeding. For this reason, it appeared that extreme poverty was the most relevant indicator to draw this line, and the World Bank International Poverty Line (living on <$1.90 per day (22), as shown in Figure 4 was used as the final filter to identify the target population. Using this filter, the target population was finally refined to 73 million children in 60 countries.

### What Is the Cost of Reaching Those Most in Need?

**Figure 5:**
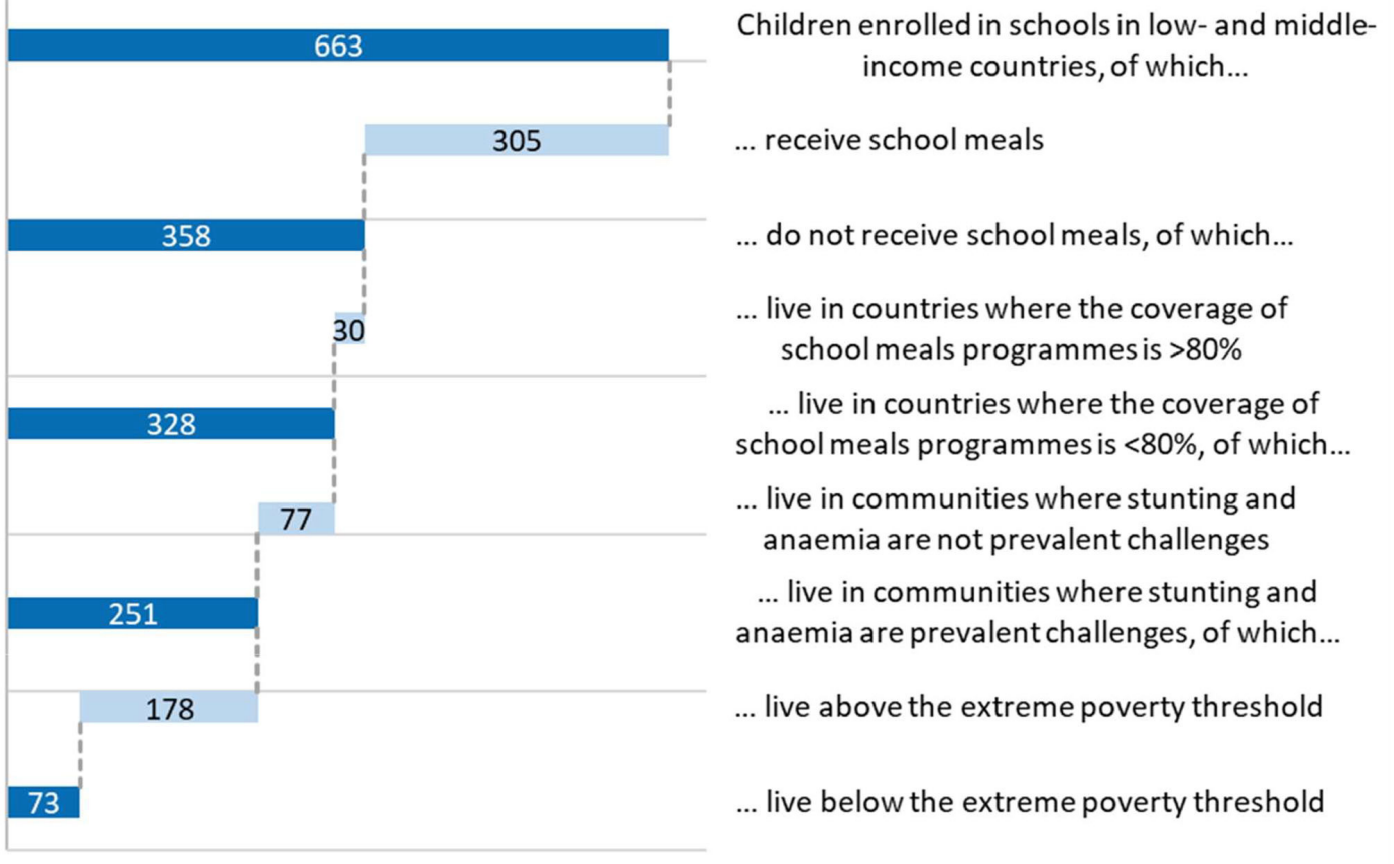
Sequential analysis for determining target population most in need.

**Figure 6:**
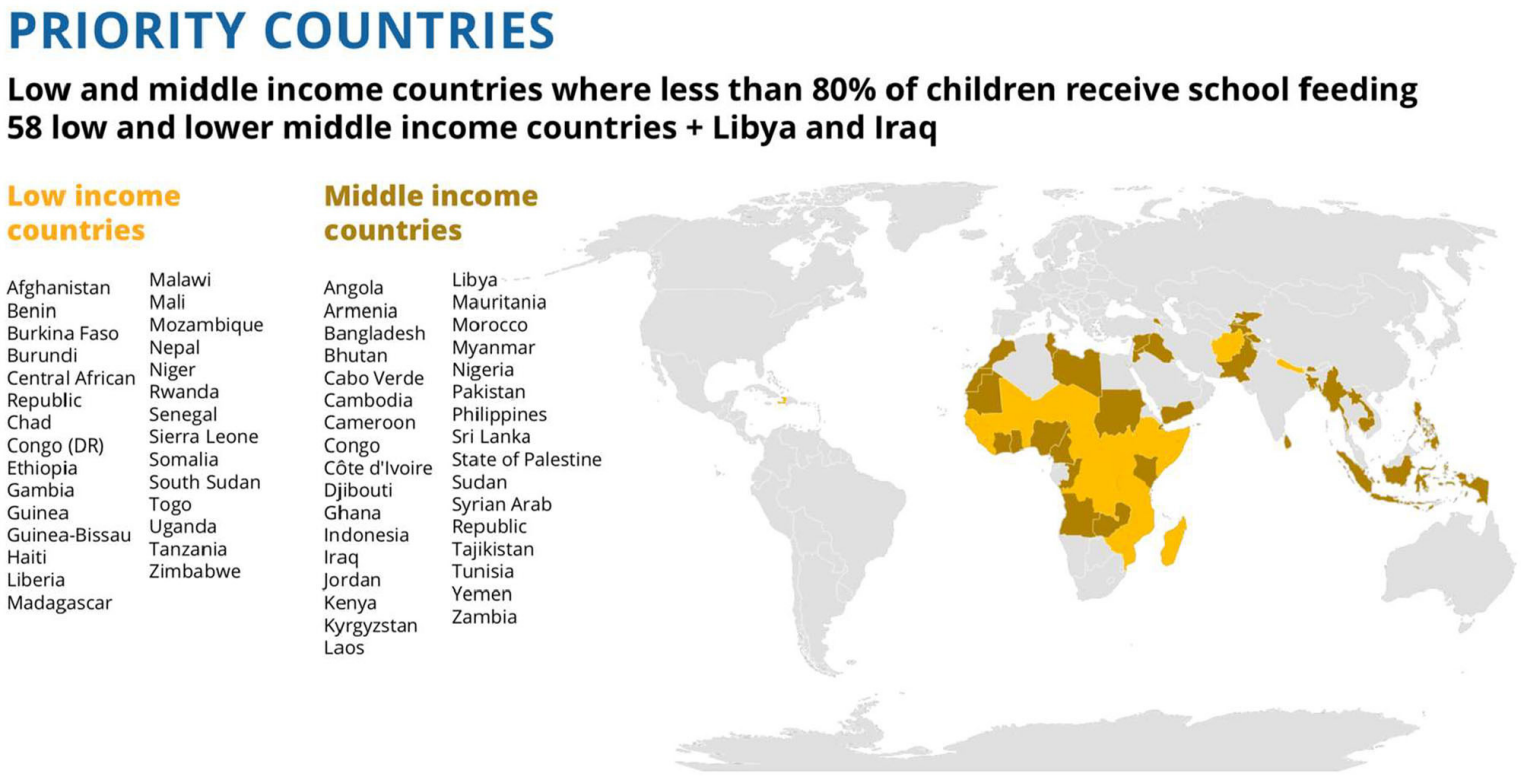
List of 60 priority countries and their geographical location.

The sequential analyses illustrated in Figure 5 below have reduced the target population to 73 million who are most in need, living in 60 countries. The list of countries and their geographical distribution is shown in Figure 6.

The cost of school feeding for the 73 million children was calculated based on published benchmark costs of providing school meals for low- and middle-income countries (see Table 2).

**Table 2:** The cost of covering 73 million children in need of school feeding is 4.7 billion USD, an average of $64 per child per year.

	**Children (millions)**	**Cost per child per year (USD)**	**School feeding (USD millions)**	**School Health Budget (USD millions)**	**Number of countries**
Middle Income Countries	26	82	2,132	618	32
Low Income Countries	47	54	2,538	507	28
Total	73	–	4,670	1,125	60

*Benchmark costs of school feeding are taken from Disease Control Priorities 3rd edition, Volume 8(1)*.

The WFP strategy recognizes that the outcome of the interventions will be optimized by the synergistic effects of a combination of school feeding and school health interventions (27, 28). The additional cost of including school health interventions was explored using the essential school health package for children from 5 to 14 years suggested in Disease Control Priorities (1, 27). These analyses are shown in Table 2, indicating an additional cost of about 20% more for the low-income package and 29% for the middle-income package, or an annual cost of USD 507 and 618 million, respectively. The total cost of the combined school feeding and school health package for the 73 million children would therefore be USD 5.8 billion annually, with around half that amount for the low income countries alone.

## Conclusions

The analysis described in this paper is the first of a sequence of studies by WFP to refine its targets for a global effort to make school feeding available to all children in need. To encourage debate and improve the quality of programs, WFP intends to share these studies in a sequence of publications.

The analysis suggests that in low- and middle-income countries globally there are some 73 million children most in need of school feeding programs, based on: not covered by national government programs; the inadequacy of current provision, the prevalence of indicators of poor nutrition, and the relative lack of financing for the countries to implement the programs themselves. Most of these children (62.7 million) are in Africa. The majority, more than 66%, live in low-income countries, but there is also a substantial minority who live in pockets of poverty in middle-income and high-middle income countries.

Addressing these needs in all 60 countries would require an extra USD 5.8 billion annually. Of this total, some USD 3 billion annually would be required to provide and resource school feeding and school health in the low-income countries alone. The additional annual amount required for middle-income countries would be some USD 2.7 billion. For these countries, it would seem probable that a substantial proportion of these resources could be made available from domestic funds. Indeed, in all cases, the logic of investing in human capital creation is that these investments in its young people today would set the country on the road to self-reliance, such that an increasing proportion of costs could be met from domestic resources. Further analyses are underway to optimize transition arrangements, including studies of successes, such as the announcement by Kenya in 2018 that the national program, established in 2006 with co-financing from WFP, was now wholly supported through domestic sources (29).

The main conclusion for now, however, is that there is currently a significant unmet need for support to school children and adolescents in low and middle-income countries, and that meeting this need is an important first step in helping a nation's young people achieve their full potential in life, and in helping these countries to increase their rank on the Human Capital Index and create economic growth (30–32). To achieve this, there is a clear need for better health and nutrition data, research, and evidence-based advocacy.

WFP is embarking on a 10 year program of support to countries, leading up to the SDG goals in 2030. The analyses described here are being used by WFP to estimate the overall scale of the response required, and so to increase the precision of planning the future allocation and procurement of new resources. These are high-level estimates, for strategic purposes. Programming at the national level continues to be based on country-level or sub-national level data, and led by the countries themselves. The DCP3 (Vol 8) school feeding edition and the global coverage numbers were launched in Tunis, 2018 by the WFP Executive Director, David Beasley. These estimates continue to inform the development of WFP's global strategy for school feeding.

WFP plans to publish further analyses, and invites comments and contributions that can help improve the quality of these analyses and the programs that result from them.

## Author Contributions

CB is the Director of the School Feeding Service of WFP, and conceived the need for this analysis to support strategic planning. LD is the Executive Director of the Partnership for Child Development (PCD) and planned and led the work. MF, NL, and KC executed the analysis. DB is a Senior Advisor to WFP and provided policy guidance. All other authors are staff or affiliates of WFP or PCD, and contributed to the execution of the analysis. The paper was drafted by DB, LD, and CB, with contributions from all authors.

## Conflict of Interest

The authors declare that the research was conducted in the absence of any commercial or financial relationships that could be construed as a potential conflict of interest.

## Footnotes

^1^Available online at: https://www.copenhagenconsensus.com
